# Community trauma exposure and post-traumatic stress disorder in Chinese children and adolescents

**DOI:** 10.3389/fpsyt.2023.1151631

**Published:** 2023-10-06

**Authors:** Ting Yuan, Xiangdong Li, Haiyang Liu, Lei-lei Guo, Jin-long Li, Guang Xu, Xiaoping Li, Lu Sun, Congzhi Wang, Liu Yang, Dongmei Zhang, Ying Hua, Yunxiao Lei, Lin Zhang

**Affiliations:** ^1^Department of Gynecology and Obstetrics Nursing, School of Nursing, Wannan Medical College, Wuhu, Anhui, China; ^2^Department of Gerontology, Yijishan Hospital, The First Affiliated Hospital of Wannan Medical College, Wuhu, Anhui, China; ^3^Student Health Center, Wannan Medical College, Wuhu, Anhui, China; ^4^Department of Surgical Nursing, School of Nursing, Jinzhou Medical University, Jinzhou, Liaoning, China; ^5^Department of Occupational and Environmental Health, Key Laboratory of Occupational Health and Safety for Coal Industry in Hebei Province, School of Public Health, North China University of Science and Technology, Tangshan, Hebei, China; ^6^Department of Radiotherapy, Third Affiliated Hospital of Jinzhou Medical University, Jinzhou, Liaoning, China; ^7^Department of Emergency and Critical Care Nursing, School of Nursing, Wannan Medical College, Wuhu, Anhui, China; ^8^Department of Internal Medicine Nursing, School of Nursing, Wannan Medical College, Wuhu, Anhui, China; ^9^Department of Pediatric Nursing, School of Nursing, Wannan Medical College, Wuhu, Anhui, China; ^10^Rehabilitation Nursing, School of Nursing, Wanna Medical College, Wuhu, Anhui, China

**Keywords:** adolescent, community, traumatic events, post-traumatic stress disorder, children

## Abstract

**Background:**

An increasing number of studies have shown the association between traumatic events occurring in childhood and adolescence and post-traumatic stress disorder (PTSD). A gap remains in the literature on the epidemiology and influencing factors of traumatic events and post-traumatic stress disorder in communities in northern China. This study aimed to determine the prevalence of traumatic events and PTSD in communities in northern China, to explore the types of stressful traumatic events and the impact of these traumatic events on children and adolescents, and to investigate the effect of sociodemographic factors on PTSD.

**Methods:**

A cross-sectional survey study was conducted among 6,027 students (7–17 years old) from 6 cities in Liaoning Province, China. The sample consisted of 2,853 males (47.34%) and 3,174 females (52.66%). The Essen Trauma-Inventory for Children and Adolescents (ETI-CA) Scale was used. The ETI-CA has 5 sections, which include type of traumatic events, worst traumatic event, post-traumatic symptoms, onset, duration, burden of PTSD, and present difficulties in different areas of life. PTSD symptoms were assessed with 23 items in Part 3 of the ETI-CA.

**Results:**

We found that 2,068 (34.3%) of 6,027 participants experienced trauma events and 686 (33.2%) of 2,068 reported PTSD. Among trauma-exposed youth (2,068), the sudden death of close relatives (33.9%), serious accidents (20.9%), and parental divorce (15.5%) were reported as the worst traumatic events. Studies have shown that after exposure to stressful life events, more than 30% of people feel nervous or upset (39.8%), scared (33.4%), helpless (32.6%), and about 10% have headaches (15.5%), rapid heartbeat (13.3%), and dizziness (11.8%). Multivariable logistic regression analyses showed that students in middle school [OR = 1.29 (1.016, 1.637)], not a student leader [OR = 0.738 (0.618, 0.881)], and their parents in single marital status significantly predicted higher PTSD prevalence the remarried [OR = 0.474 (0.252, 0.893)], married [OR = 0.42 (0.227, 0.778)].

**Conclusion:**

The present study suggests the government to train psychological counselors in schools and communities to provide emotional and psychological support, as well as the school leaders and parents to elevate adolescents' psychological *suzhi*. Particularly, counseling and professional support should be given to those students whose parents are single.

## 1. Introduction

The Diagnostic and Statistical Manual of Mental Disorders, fourth edition (DSM-IV), proposed traumatic events as exposure to one or more of the following events by personal experience, witnessing, repetition of distasteful details, or extreme experiences, involving actual death or threat of death, serious injury, or threat to the physical integrity of self or others, and intense fear, helplessness, or horror response (1). The second part of the definition in the DSM-V has been eliminated (2).

Exposure to traumatic events at least once was common in childhood and adolescence. It was reported that 63.0% of the children and 89.5% of the adolescents had ever experienced traumatic events in Linköping, Sweden (3), 60.0% of adolescents in the United States (4), 68.9% of adolescents in Mexican (5), 83.7% of children and adolescents in the Gaza Strip, Palestinian (6), and nearly all adolescents (99.7%) in Soweto, South Africa (7). “Children of Rural-Urban Chinese Migrants” reported that 47.1% had experienced traumatic events (8).

Traumatic events that occurred during childhood and adolescence increased susceptibility to mental disorders (9) [post-traumatic stress disorder, depression (10), and anxiety (11)], to adverse physical outcomes [hippocampal structural changes (12), amygdala connectivity altered (13), and developing psychopathology (4)], and to cause a variety of consequences, such as increasing the risk of suicidality (14), aggression behaviors directed toward the self and others (15), and substance abuse (16, 17).

Post-traumatic stress disorder (PTSD) is a psychiatric disorder that is originally triggered after a traumatic life event (18, 19), including natural disasters [e.g., earthquakes (20, 21), floods (22), tsunamis (23), and tornadoes (24)], injuries [e.g., physical abuse (25), sexual assaults (26), and vehicle accidents (27)], violence (28) [e.g., wars (6), terrorist attacks (29), and interpersonal crimes (15)], and deaths (the deaths of an intimate one and life-threatening illnesses such as cancer). The main symptoms of PTSD are intrusion or re-experiencing, avoidance, hyper-arousal, and negative alterations in cognition and mood.

Most literature about traumatized children and adolescents came from disasters, violent injuries, war combat, and death. Approximately 11–72.8% of traumatized adolescents may develop PTSD as a result of natural disasters (20, 22). Approximately 37–50% of adolescents who were exposed to physical abuse and sexual assault have met the criteria for a diagnosis of PTSD (30, 31). The proportion of war-related traumatized adolescents who meet the diagnostic criteria for PTSD is as high as 36–53% (6, 32, 33). Traumatic events are prevalent in children and adolescents. However, traumatized children and adolescents lack effective skills to cope with traumatic events and are more likely to develop PTSD. The population of children in China reached 246 million in 2021, which is approximately accounted for one-tenth of the world's child population (34, 35). The rate of PTSD was 6.7–9.7%, among Chinese rural children and adolescents with traumatic experiences (8, 36). The epidemiological characteristics of traumatic events and PTSD among children and adolescents in communities of China are still unclear.

PTSD followed by Trauma exposures during early childhood could impair child development, disrupt attachment security and self-regulatory processes, and lead to poor physical and mental health outcomes in childhood and across the lifespan (37). It has been shown that adolescents with PTSD are at increased risk for additional mental health problems, including self-harm, suicide attempts, social isolation, loneliness, and functional impairments (38). The frontolimbic circuits of youth with PTSD exhibit developmental and overt abnormalities, which may contribute to an increase in threat reactivity and a decline in emotion regulation (39). PTSD in adolescence is a risk factor for long-term psychopathology, substance abuse, and quality of life deficits (40, 41).

Demographic factors [e.g., age (42–44), female gender (45, 46), not living with parents (47, 48), living in a rural area (49), poor family functioning (50, 51), and trauma exposure history] and socioeconomic factors (e.g., low economic resources and low social support) play a role in the risk of PTSD (42, 52). Age was a contradictory factor. Both youth and older age were thought to be associated with an increased likelihood of presenting with symptoms of PTSD (42–44).

The study aimed to explore the types of traumatic events experienced, to evaluate the rates of probable post-traumatic stress disorder, and to investigate predictors of PTSD symptoms in a sample of 6,027 children and adolescents in Liaoning Province, China. We hypothesized that risk factors such as demographic variables (including education level, age, gender, grade level, whether living with parents, whether student leaders, living area, living city, parent's marital status, parent's education level, annual household income) might be associated with PTSD in Chinese children with traumatic experiences in Liaoning Province.

## 2. Materials and methods

### 2.1. Study participants

This study sample was recruited from 6,644 children and adolescents living in Liaoning, a northeastern province in China. If a student submitted a questionnaire with missing data, the data was excluded (*n* = 364). Research ethics were also considered, and if a student did not submit informed consent due to parental disapproval, the study was excluded (*n* = 253). Finally, the sample was 6,027, and the response rate was 90.7%.

Demographic characteristics consisted of city, education, age, gender, grade, living with their parents, student leaders, living area, marital status of parents, educational level of parents, and annual household income. Six cities were selected for this study, including Shenyang, Dalian, Jingzhou, Yingkou, Liaoyang, and Dandong. Elementary and middle school students were included, from grades 3 to 9. Age was grouped as 7–10 years old, 11–13 years old, and 14–17 years old. The majority of participants were female (*n* = 3,174, 52.7%). Almost all students lived with their parents (*n* = 5,430, 90.1%). Nearly half of the students were student leaders (*n* = 2,606, 43.2%). About three-quarters of the students lived in urban areas (*n* = 4,637, 76.9%). The marital status of parents was categorized into three groups: single, remarried, and married. Parents' educational level includes less than high school, vocational school, college, and master's degree and above. Annual household income was categorized as <$3,251, $3,251 to $8,127, $8,127 to $16,254, $16,254 to $32,508, and >$32,508.

### 2.2. Sample size

Based on the pilot study, we calculated the sample size. Prior to the survey, questionnaires were randomly distributed to 400 students in an elementary school and a secondary school in Jinzhou City. In the pilot study, the prevalence of PTSD (*p*) was 33.3%. Using the following formula to calculate the sample size, α values were considered to be 0.01 and *d* was considered to be 0.10 × *p*. The intended sample size we needed for the study would have been 1,333. Finally, we recruited 6,027 participants (53).


$$\begin{array}{l} n\;=\;\frac{Z_{1-\frac{\alpha }{2}}^{2}\times p\left(1-p\right)}{d^{2}} \end{array}$$

### 2.3. Procedure

**Figure 1:**
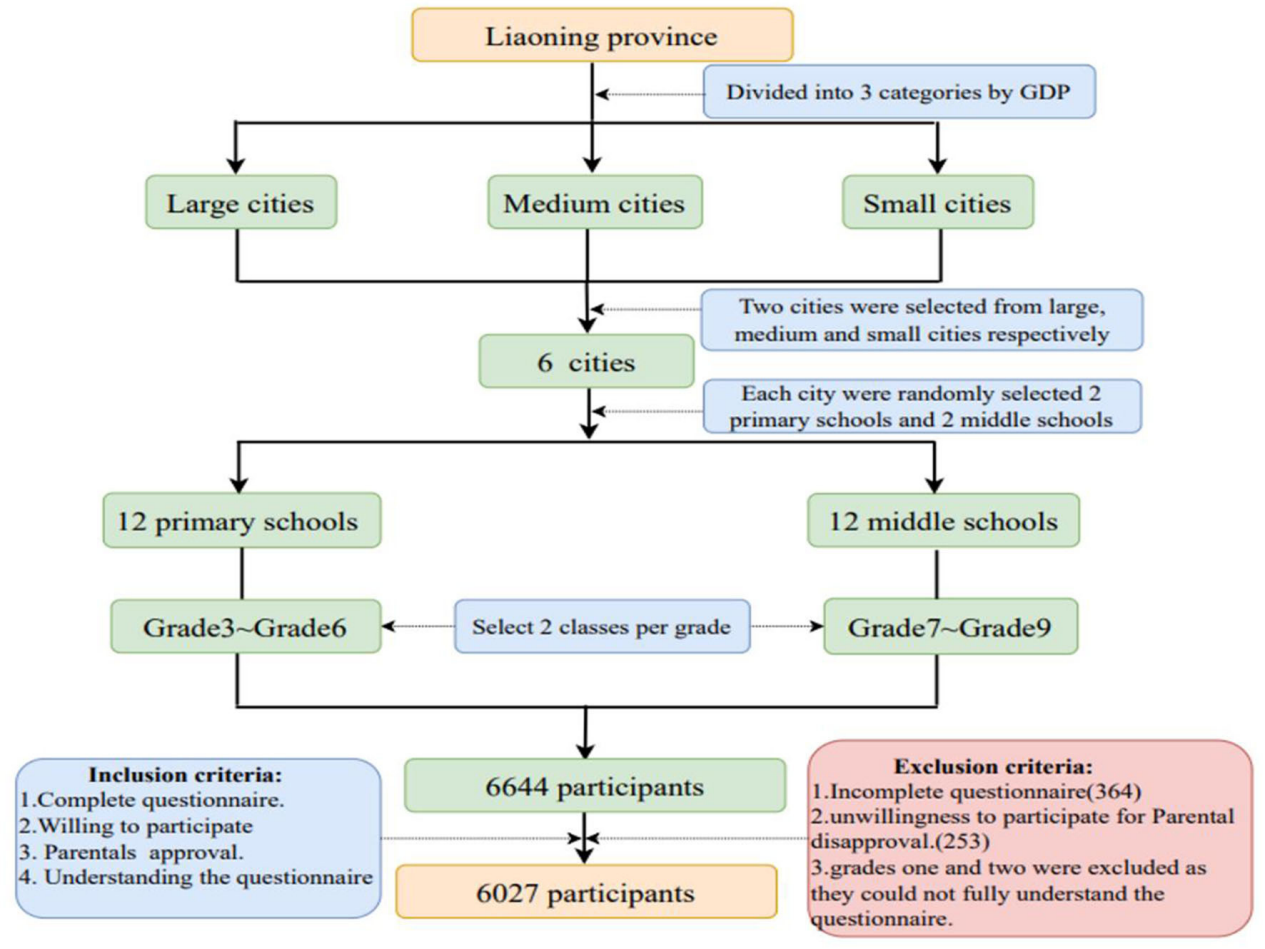
Participant and data collection flow chart.

The research team assessed trauma exposure and PTSD in children and adolescents in Liaoning through a cross-sectional survey using questionnaires, from 1 June to 31 December 2013. Six cities were chosen to provide a diverse sample based on the gross domestic product (GDP) ranking. There were Shenyang, Dalian, Yingkou, Jinzhou, Liaoyang, and Dandong. Before starting the investigation, teachers handed out informed consent forms to students after the safety education session. The students took the informed consent forms home to their parents. Parents who agreed to the student's participation in the survey signed the informed consent form. The next day, teachers distributed questionnaires to students who volunteered to participate in the study and informed consent was obtained from parents. All children were informed that the questionnaire was anonymous, and their answers were strictly confidential. Researchers and trained teachers provided assistance to children and young people in completing the questionnaire and explaining any questions during the survey. All the researchers involved in the survey were trained by child psychologists. If a participant meets the criteria for PTSD or is highly symptomatic, the psychology teacher will provide psychological intervention services. Multistage stratified cluster random sampling was adopted to investigate the prevalence of traumatic events and post-traumatic stress disorder (PTSD) and the factors associated with PTSD in children and adolescents in northern Chinese communities. Researchers and trained teachers provide assistance to children and adolescents with problems during the survey. Participants and data collection procedure are presented in Figure 1.

### 2.4. Ethical considerations

This study was conducted according to the principles of the Declaration of Helsinki. All participants provided an informed consent form signed by their parents, giving their consent for the students to participate in the survey. The study was approved by the Ethics Committee of the Nursing Department of Liaoning Medical College (Approval Number 2013–00).

### 2.5. Assessment instruments

#### 2.5.1. Demographic questionnaire

Data are presented for age, sex, grade, living area, education, living situation, student leaders, living area, marital status of parents, educational level of parents, and the annual income of parents. The demographics of the study participants are presented in Table 1.

**Table 1:** Demographic variables frequencies of the participants (*N* = 6,027).

**Variables**	**Categories**	***n* (%)**
City	Shenyang	918 (15.2)
	Dalian	1,006 (16.7)
	Jingzhou	1,121 (18.6)
	Yingkou	924 (15.3)
	Liaoyang	1,001 (16.6)
	Dandong	1,057 (17.5)
Education	Elementary school student	3,199 (53.1)
	Middle school student	2,828 (46.9)
Age (years)	7~10	1,005 (16.7)
	11~13	3,633 (60.3)
	14~17	1,389 (23.1)
Gender	Boy	2,853 (47.3)
	Girl	3,174 (52.7)
Grade	3	656 (10.9)
	4	825 (13.7)
	5	787 (13.1)
	6	931 (15.5)
	7	988 (16.4)
	8	1,027 (17.0)
	9	813 (13.5)
Living with their parents	No	597 (9.9)
	Yes	5,430 (90.1)
Student leaders	No	3,421 (56.8)
	Yes	2,606 (43.2)
Living area	Rural	1,390 (23.1)
	Urban	4,637 (76.9)
Marital status of parents	Single	112 (1.9)
	Remarried	1,291 (21.4)
	Married	4,624 (76.7)
Educational level of parents	Less than high school	3,500 (58.1)
	Vocational school	1,441 (23.9)
	College	918 (15.2)
	Master's degree or above	168 (2.8)
Annual household income ($)	< 3,251	1,506 (25.0)
	3,251~8,127	2,501 (41.5)
	8,127~16,254	1,473 (24.4)
	16,254~32,508	400 (6.6)
	>32,508	147 (2.4)

Student leaders, usually the leaders of a class, are usually elected by the class and are also known as class representatives, peer mentors, and teacher's assistants, just like the class president, which will help them develop leadership skills.

#### 2.5.2. Traumatic experiences and PTSD

Traumatic experiences and PTSD symptoms were assessed with Essen Trauma-Inventory for Children and Adolescents (ETI-CA) developed by Tagay S et al. (54). Ruifang Jiang studied and validated the reliability and validity of the Chinese version of the Child and Adolescent Trauma Inventory (ETI-CA). The results show that the Chinese version of the ETI-CA is a good tool for assessing trauma symptoms in children and adolescents (55).

The ETI-CA has five sections, which include type of traumatic event, worst traumatic event, post-traumatic symptoms, onset, duration, burden of PTSD, and present difficulties in different areas of life. PTSD symptoms were assessed with 23 items in Part 3 of the ETI-CA.

##### 2.5.2.1. Types of traumatic events

This section mainly consists of identifying the type of trauma experienced by the student and whether they have ever experienced a traumatic event personally or as a witness. The types of trauma reported are categorized as follows: natural disaster, severe accident, severe illness or injury, assault by a stranger or a familiar person, sudden death of a close family member, imprisonment, sexual abuse, war trauma, neglect, and other stressful events. It contains 12 items.

##### 2.5.2.2. The worst traumatic event

On the Traumatic Events Checklist listed in Section 1, which event was their worst experience? If the event is not included, they can override another event. The timing and subjective perception of the most severe traumatic event were also recorded. These are reported by items 13–15.

##### 2.5.2.3. The post-traumatic symptoms

This part of the instrument consists of 23 items investigating participants' post-traumatic symptoms over the past 4 weeks, each item using a 4-point Likert scale ranging from “not at all” (0 points) to “very often” (3 points). The total score ranged from 0 to 69, with 16–26 and ≥27 representing suspected and confirmed PTSD, respectively. Higher scores indicate more severe symptoms. The Cronbach's alpha coefficient of this scale is 0.904. These items correspond to the DSM-IV criteria for Post-Traumatic Stress Disorder.

In addition, the 24th item asked respondents to identify which physical complaints they had experienced. These included stomach aches, headaches, sickness, diarrhea, tremor, dizziness, racing heartbeat, breathlessness, and seizures.

##### 2.5.2.4. The onset, duration, and burden of PTSD

The fourth part consists of 3 items. The questions were about the onset, duration, and burden of their post-traumatic symptoms under the influence of the most severe traumatic event. How burdensome this event is to you right now, on a 6-point Likert scale ranging from 0 = “not at all” to 6 = “extremely.”

##### 2.5.2.5. The impact of traumatic events in different areas of life

This section asked how difficult the student is currently in different areas. These areas include school/employment, household chores and duties, hobbies, and leisure activities, relationships with friends, relationships with family members, and sexuality. The answers to these questions are based on a 4-point Likert scale ranging from 0 = “none” to 3 = “strong”.

### 2.6. Statistical analysis

Data were analyzed by IBM SPSS version 21.0 (Chicago, IL, United States). Frequencies and percentages were calculated for the categorical data. *T*-tests and one-way ANOVA were used to investigate the difference in the continuous variables between groups. The chi-square test was used to verify differences in the categorical variables between groups. Univariate and multivariate logistic regression analyses were used to explore the impact of factors on post-traumatic stress disorders. A two-sided *P*-value < 0.05 was considered statistically significant.

## 3. Results

### 3.1. Demographic characteristics

The sample size of the survey was 6,027, which consisted of 52.7% (*n* = 3,174) girls and had a mean age of 12.3 years (range from 7 to 17 years, SD = 1.62). Most of the students were living in rural (76.9%, *n* = 4,637) and lived with their parents (90.1%, *n* = 5,430). The demographics of the study are presented in Table 1.

### 3.2. Prevalence of traumatic events and levels of total PTSD

Of the 6,027 samples, the number of people who experienced different traumatic events was 2,068 (34.3%) during their life. The mean PTSD score for the sample that had experienced a traumatic event was 20.30 ± 13.86.

T-tests indicated that middle school students reported higher PTSD scores than elementary school students (*P* < 0.001). Students who lived with their parents scored significantly lower on PTSD than those who did not live with them (*P* < 0.05). Compared to those who were student leaders, students who were not student leaders scored higher on PTSD (*P* < 0.05). Those living in rural areas reported higher PTSD scores than those living in urban areas (*P* < 0.001) (Table 2).

**Table 2:** Frequencies of trauma events and levels of total PTSD (*N* = 2,068).

**Variables**	**Categories**	***n* (%)**	**Mean ±SD**	***P***
City	Shenyang	305 (5.1)	19.46 ± 14.42	< 0.001
	Dalian	320 (5.3)	20.47 ± 13.64	
	Jingzhou	343 (5.7)	19.24 ± 13.86	
	Yingkou	341 (5.7)	21.72 ± 13.98	
	Liaoyang	458 (7.6)	18.54 ± 12.85	
	Dandong	301 (5.0)	22.99 ± 14.38	
Education	Elementary school student	1,017 (16.9)	18.72 ± 13.38	< 0.001
	Middle school student	1,051 (17.4)	21.82 ± 14.15	
Age (years)	7~10	3,006 (49.9)	15.64 ± 12.30	< 0.001
	11~13	1,286 (21.3)	20.77 ± 13.85	
	14~17	482 (8.0)	21.92 ± 14.22	
Gender	Boy	997 (16.5)	20.34 ± 14.02	0.884
	Girl	1,071 (17.8)	20.25 ± 13.71	
Grade	3	173 (2.9)	17.47 ± 13.22	< 0.001
	4	240 (4.0)	18.12 ± 13.66	
	5	287 (4.8)	19.28 ± 13.25	
	6	317 (5.3)	19.25 ± 13.36	
	7	401 (6.7)	21.61 ± 14.12	
	8	367 (6.1)	22.86 ± 13.88	
	9	283 (4.7)	20.77 ± 14.48	
Living with their parents	No	238 (4.0)	22.13 ± 13.19	0.030
	Yes	1,830 (30.4)	20.06 ± 13.93	
Student leaders	No	1,080 (52.2)	20.92 ± 14.12	0.032
	Yes	988 (47.8)	19.61 ± 13.54	
Marital status of parents	Single	58 (1.0)	26.60 ± 14.48	0.001
	Remarried	530 (8.8)	20.54 ± 13.27	
	Married	1,480 (24.6)	19.96 ± 13.99	
Living area	Rural	465 (7.7)	22.32 ± 14.23	< 0.001
	Urban	1,603 (26.6)	19.71 ± 13.70	
Education status of parents	Less than high school	1,207 (19.2)	20.50 ± 14.01	0.181
	Vocational school	530 (8.8)	20.58 ± 14.11	
	College	286 (4.8)	19.51 ± 12.80	
	Master's degree or above	45 (0.8)	16.44 ± 13.03	
Annual household income ($)	< 3,251	504 (8.4)	20.81 ± 14.73	0.766
	3,251~8,127	883 (14.7)	20.08 ± 13.49	
	8,127~16,254	493 (8.2)	19.90 ± 13.81	
	16,254~32,508	138 (2.3)	21.16 ± 13.74	
	>32,508	50 (0.8)	20.42 ± 12.17	

One-way ANOVA indicated that students in Yingkou and Dandong reported higher PTSD scores than in other cities (*P* < 0.001). The oldest age group (14 to 17 years) scored significantly higher on PTSD than the older age group (*P* < 0.001). Compared to students in the lower grades, those in the upper grades (Grades 7–9) scored higher on PTSD (*P* < 0.001). Students whose parents were single reported higher PTSD scores than those whose parents were remarried and married (*P* < 0.01) (Table 2).

### 3.3. Distribution of different types of traumatic events

Table 3 shows different types of traumatic events students experience during their life. Of the 2,068 sample, the three worst traumatic events reported were the sudden death of close relatives (33.9%), serious accidents (20.9%), and parental divorce (15.5%). The three types of traumatic events witnessed by all students with the highest frequency were serious accidents (31.6%), the sudden death of close relatives (16.9%), and severe illness (14.0%). Regarding personally experienced trauma, the three types of traumatic events that all students experienced most were the sudden death of close relatives (19.3%), parental divorce (10.0%), and neglect (9.3%). The most severe interpersonal trauma experienced by girls was the same as the overall sample. The frequency of traumatic events in the three most severe personal experiences reported by boys was the sudden death of a close family member (14.3%), other stressful events (9.9%), and severe illness/injury (9.5%).

**Table 3:** Different types of traumatic events students experienced during their life (*N* = 2,068).

	**In-person** ***n*** **(%)**			**Witnessed** ***n*** **(%)**			**Your worst experience** ***n*** **(%)**		
**Item**	**Boy**	**Girl**	**Total**	**Boy**	**Girl**	**Total**	**Boy**	**Girl**	**Total**
1. Natural disasters (e.g., flood, thunderstorm, earthquake)	90 (9.0)	65 (6.1)	155 (7.5)	134 (13.4)	94 (8.8)	228 (11.0)	108 (10.8)	78 (7.3)	186 (9.0)
2. Serious accident, fire, or explosion (e.g., car, industrial, plane, or boating accident)	95 (9.5)	67 (6.3)	162 (7.8)	296 (29.7)	358 (33.4)	654 (31.6)	199 (20.0)	234 (21.9)	433 (20.9)
3. Severe illness/injury (e.g., stroke, cancer, heart attack, severe surgery)	50 (5.0)	43 (4.0)	93 (4.5)	138 (13.8)	151 (14.1)	289 (14.0)	87 (8.7)	79 (7.4)	166 (8.0)
4. Assault by a stranger (e.g., being physically attacked, robbed, threatened with a gun)	26 (2.6)	19 (1.8)	45 (2.2)	79 (7.9)	43 (4.0)	122 (5.9)	28 (2.8)	11 (1.0)	39 (1.9)
5. Assault by a family member or someone you know (e.g., being physically attacked, robbed, threatened with a gun)	63 (6.3)	55 (5.1)	118 (5.7)	32 (3.2)	39 (3.6)	71 (3.4)	43 (4.3)	26 (2.4)	69 (3.3)
6. Sudden death or loss of a close person, or family member (e.g., by accident, suicide, or murder)	143 (14.3)	257 (24.0)	400 (19.3)	166 (16.7)	184 (17.2)	350 (16.9)	294 (29.5)	407 (38.0)	701 (33.9)
7. Imprisonment (e.g., prison inmate, prisoner of war, hostage)	13 (1.3)	12 (1.1)	25 (1.2)	18 (1.8)	10 (0.9)	28 (1.4)	9 (0.9)	0	9 (0.4)
8. Sexual abuse by a stranger (e.g., unwanted or forced sexual contact, rape)	11 (1.1)	15 (1.4)	26 (1.3)	3 (0.3)	10 (0.9)	13 (0.6)	0	11 (1.0)	11 (0.5)
9. Sexual abuse by a family member or someone you know (e.g., unwanted or forced sexual contact, rape)	11 (1.1)	12 (1.1)	23 (1.1)	3 (0.3)	1 (0.1)	4 (0.2)	0	1 (1.0)	1 (0.1)
10. Stay in a war zone	12 (1.2)	12 (1.1)	24 (1.2)	7 (0.7)	5 (0.5)	12 (0.6)	0	2 (0.2)	2 (0.1)
11. Neglect (e.g., constant rejection, not enough parental care)	93 (9.3)	100 (9.3)	193 (9.3)	33 (3.3)	34 (3.2)	67 (3.2)	67 (6.7)	63 (5.9)	130 (6.3)
12. Other stressful events (Leave your parents or family, parent divorce)	99 (9.9)	107 (10.0)	206 (10.0)	48 (4.8)	28 (2.6)	76 (3.7)	162 (16.3)	159 (14.9)	321 (15.5)

A significant gender difference was found for personally experienced traumatic events 1, 2, 6.

A significant gender difference was found for witnessed traumatic events 1, 4.

A significant gender difference was found for the worst experiences of trauma 1, 4, 5, 6, 7, 8.

**Figure 2:**
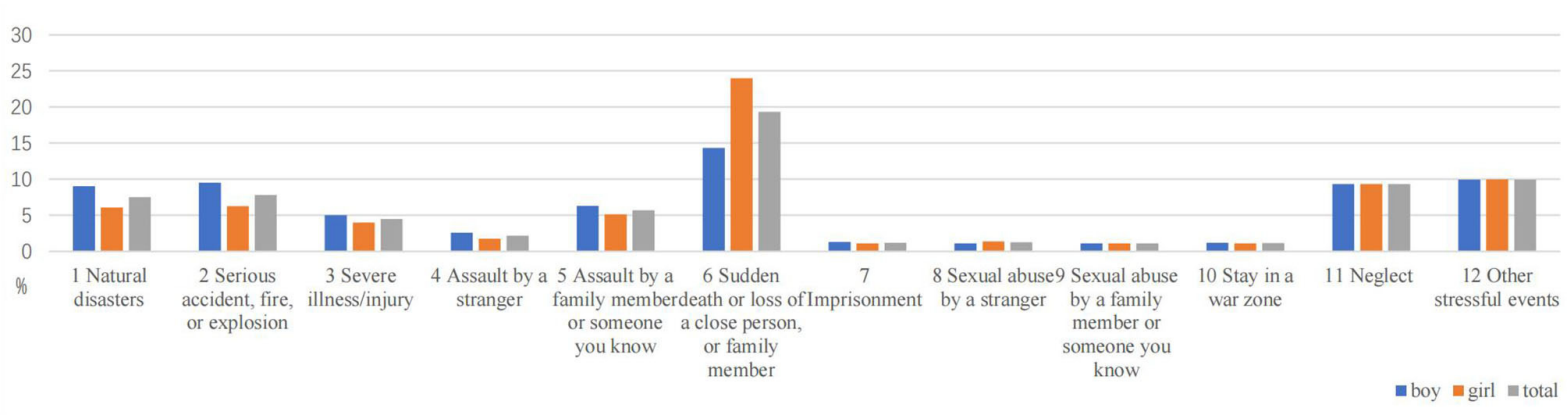
Gender differences in personally experienced traumatic events.

**Figure 3:**
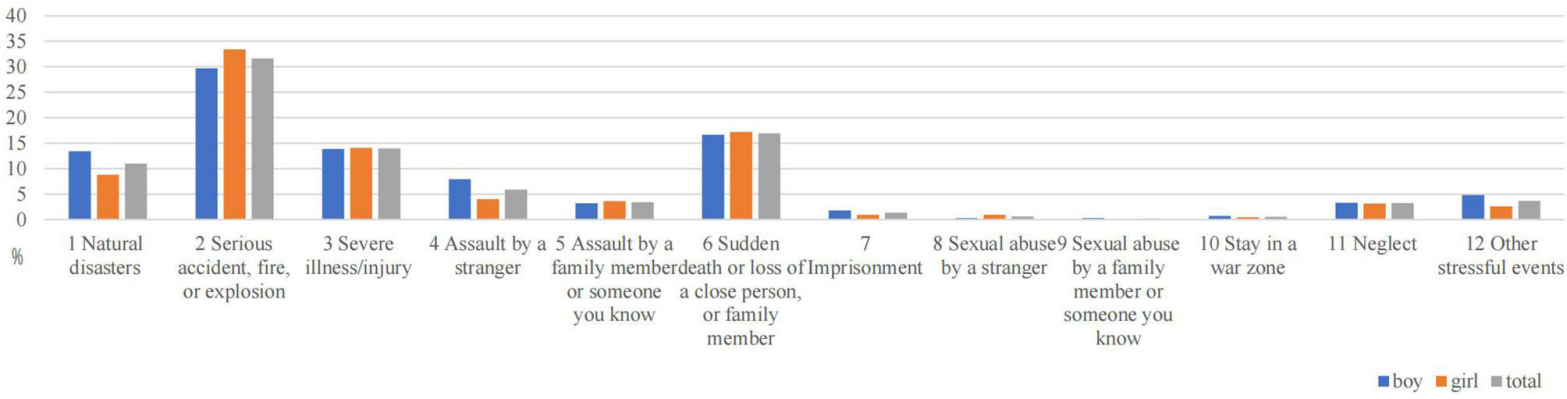
Gender differences in witnessed traumatic events.

**Figure 4:**
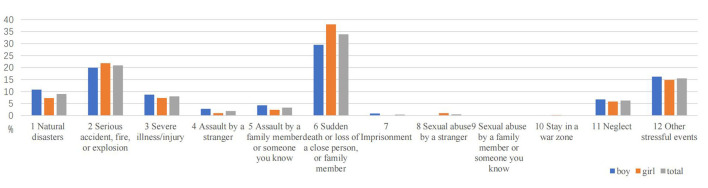
The worst experience of traumatic events in different genders.

Among personally experienced traumatic events, there were statistically significant differences between boys and girls mainly in natural disasters, serious accidents, sudden death, or loss of a close person (*P* < 0.05). Among the traumatic events witnessed, a significant gender difference was found between boys and girls for traumatic events in natural disasters and assault by a stranger (*P* < 0.05). There were significant gender differences in the following worst traumatic events experienced: natural disasters, assault by a stranger, assault by a family member or someone you know, sudden death or loss of a close person, imprisonment, and sexual abuse by a stranger (*P* < 0.05). Details are shown in Table 3 and Figures 2–4.

### 3.4. Distribution of feelings—physical complaints—burden in the worst event

In the worst event, more than 30 % of respondents reported feeling very tense or restless (39.8%), scared (33.4%), helpless (32.6%), and physically injured themselves (31.5%). The main physical discomfort experienced by the participants was headache (15.5%), rapid heartbeat (13.3%), and dizziness (11.8%). After a stressful life event, approximately 5 % of adolescents felt strongly extremely or strongly burdened whereas 20% felt little or no impact from the traumatic event (Table 4).

**Table 4:** Distribution of feelings—physical complaints—burden in the worst event (*N* = 2,068).

**The feelings**		**The physical complaints**		**The severity of the burden**	
**Item**	***n*** **(%)**	**Item**	***n*** **(%)**	**Item**	***n*** **(%)**
Yourself physically injured	412 (19.9)	Stomach aches	181 (8.8)	Not at all	560 (27.1)
Life was at risk	135 (6.5)	Headaches	320 (15.5)	Very slightly	721 (34.9)
Someone else physically injured	652 (31.5)	Sickness	155 (7.5)	Slightly	420 (20.3)
Someone else's life was at risk	435 (21.0)	Diarrhea	64 (3.1)	Moderately	140 (6.8)
Feel helpless	675 (32.6)	Tremor	75 (3.6)	Strongly	116 (5.6)
Extremely scared	690 (33.4)	Dizziness	244 (11.8)	Extremely	111 (5.4)
Feel very tense or restless	822 (39.8)	Racing heartbeat	274 (13.3)		
		Breathlessness	111 (5.37)		
		Seizures	28 (1.35)		

### 3.5. Current difficulties in different areas of life

Adolescents with a history of trauma seemed to be more prone to experience difficulties at school and in doing household chores, compared to hobbies and leisure activities, relationships with friends, relationships with family members, and sexuality. The fewest students reported having difficulties with sexuality (Table 5).

**Table 5:** Current difficulties in different areas of life (*N* = 2,068).

**Item**	**None**	**Slight**	**Moderate**	**Strong**
School/employment	1,395 (67.5)	451 (21.8)	168 (8.1)	54 (2.6)
Household chores and duties	1,376 (66.5)	443 (21.4)	190 (9.2)	59 (2.9)
Hobbies and leisure activities	1,549 (74.9)	318 (15.4)	154 (7.5)	47 (2.3)
Relationships to friends	1,549 (74.9)	336 (16.3)	127 (6.1)	56 (2.7)
Relationships with family members	1,630 (78.8)	264 (12.8)	115 (5.6)	59 (2.9)
Sexuality	1,793 (86.7)	166 (8.0)	52 (2.5)	57 (2.8)

### 3.6. Traumatic events and frequency of PTSD

**Figure 5:**
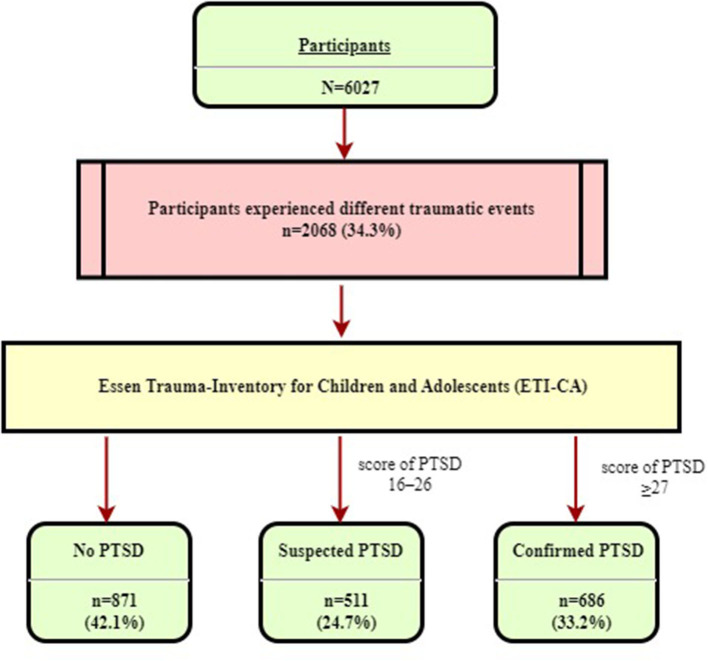
Frequencies of traumatic events and PTSD.

A chi-square analysis was performed to explore demographic differences between participants with total PTSD and those without PTSD. In the overall sample, the prevalence of confirmed PTSD was 11.4% (686 of 6,027) and the prevalence of suspected PTSD was 8.5% (511 of 6,027). Among traumatized participants, the prevalence of confirmed PTSD was 33.2% (686 out of 2,068) and the prevalence of suspected PTSD was 24.7% (511 out of 2,068) (Table 6 and Figure 5).

**Table 6:** Chi-square tests to explore demographic differences between participants with total PTSD and without PTSD (*N* = 2,068).

**Variables**	**Categories**	**Suspected PTSD (511)**	**Confirmed PTSD (686)**	**Total**	**NO**	***χ2***	***df***	***P***
				**PTSD (1,197)**	**PTSD (871)**			
		***n* (%)**	***n* (%)**	***n* (%)**	**n (%)**			
City	Shenyang	64 (12.5)	98 (14.3)	162 (13.5)	143 (16.4)	14.331	5	0.014
	Dalian	86 (16.8)	104 (15.2)	190 (15.9)	130 (14.9)			
	Jingzhou	88 (17.2)	97 (14.1)	185 (15.5)	158 (18.1)			
	Yingkou	81 (15.9)	131 (19.1)	212 (17.7)	129 (14.8)			
	Liaoyang	136 (26.6)	118 (17.2)	254 (21.2)	204 (23.4)			
	Dandong	56 (11.0)	138 (20.1)	194 (16.2)	107 (12.3)			
Education	Elementary school student	248 (48.5)	290 (42.3)	538 (45.0)	479 (55.0)	20.368	1	< 0.001
	Middle school student	263 (51.5)	396 (57.7)	659 (55.1)	392 (45.0)			
Age (years)	7~10	76 (14.9)	61 (8.9)	137 (11.5)	163 (18.7)	23.461	2	< 0.001
	11~13	312 (61.1)	446 (65.0)	758 (63.3)	528 (60.6)			
	14~17	123 (24.1)	179 (26.1)	302 (25.2)	180 (20.7)			
Sex	Boy	252 (49.3)	325 (47.4)	577 (48.2)	420 (48.2)	0.000	1	0.994
	Girl	259 (50.7)	361 (52.6)	620 (51.8)	451 (51.8)			
Grade	3	44 (8.6)	44 (6.4)	88 (7.4)	85 (9.8)	26.303	6	< 0.001
	4	59 (11.6)	66 (9.6)	125 (10.4)	115 (13.2)			
	5	69 (13.5)	88 (12.8)	157 (13.1)	130 (14.9)			
	6	76 (14.87)	92 (13.4)	168 (14.0)	149 (17.1)			
	7	87 (17.03)	158 (23.0)	245 (20.5)	156 (17.9)			
	8	94 (18.4)	153 (22.3)	247 (20.6)	120 (13.8)			
	9	82 (16.1)	85 (12.4)	167 (14.0)	116 (13.3)			
Living with their parents	No	64 (12.5)	87 (12.7)	151 (12.6)	87 (10.0)	3.415	1	0.065
	Yes	447 (87.5)	599 (87.3)	1,046 (87.4)	784 (90.0)			
Student leaders	No	282 (55.2)	379 (55.3)	661 (55.2)	419 (48.1)	10.231	1	0.001
	Yes	229 (44.8)	307 (44.8)	536 (44.8)	452 (51.9)			
Living area	Rural	102 (20.0)	193 (28.1)	295 (24.6)	170 (19.5)	7.604	1	0.006
	Urban	409 (80.0)	493 (71.9)	902 (75.4)	701 (80.5)			
Marital status of parents	Single	15 (2.9)	29 (4.3)	44 (3.7)	14 (1.6)	11.309	2	0.004
	Remarried	125 (24.5)	197 (28.7)	322 (26.9)	208 (23.9)			
	Married	371 (72.6)	460 (67.1)	831 (69.4)	649 (74.5)			
Education status of parents	Less than high school	295 (14.3)	417 (20.2)	712 (59.5)	495 (56.8)	5.396	3	0.145
	Vocational school	122 (5.9)	179 (8.7)	301 (25.1)	229 (27.1)			
	College	87 (4.2)	78 (3.8)	165 (13.8)	121 (14.3)			
	Master's degree or above	7 (0.3)	12 (0.6)	19 (1.6)	26 (3.1)			
Annual household income ($)	< 20,000	116 (22.7)	178 (26.0)	294 (24.6)	210 (24.1)	0.205	4	0.995
	20,000~50,000	223 (43.6)	285 (41.6)	508 (42.4)	375 (43.1)			
	50,000~100,000	126 (24.7)	160 (23.3)	286 (23.9)	207 (23.8)			
	100,000~200,000	32 (6.3)	47 (6.9)	79 (6.6)	59 (6.8)			
	>200,000	14 (2.7)	16 (2.3)	30 (2.5)	20 (2.3)			

Total PTSD = Suspected PTSD +Confirmed PTSD.

Total PTSD (score of PTSD ≥ 16).

Suspected PTSD (score of PTSD = 16–26).

Confirmed PTSD (score of PTSD ≥ 27).

The differences among students with total PTSD and without PTSD were observed in the distribution of cities (χ^2^ = 14.331, *P* = 0.014). Students in Dandong and Yingkou reported more PTSD than those in Shenyang, Dalian, Jingzhou, and Liaoyang. The frequency of PTSD diagnoses also differed significantly among students with different levels of education (χ^2^ = 20.368, *P* < 0.001). Middle school students were more likely to meet the PTSD criteria than primary school students. Significant differences were also found between age and PTSD (χ^2^ = 23.461, *P* < 0.001). Students in the 14–17 age group were more likely to meet the criteria for PTSD than children in the 7–10 and 11–13 age groups. The grade is a significant influence on PTSD (χ^2^ = 26.303, *P* < 0.001). Students in grades 7–9 were more often diagnosed with PTSD than students in grades 3–6. The difference in the living area was found to have a significant effect on PTSD (χ^2^ = 7.604, *P* = 0.006), and students who lived in rural were more likely to develop PTSD than those who lived in urban. Furthermore, student leaders were associated with PTSD (χ^2^ = 10.231, *P* < 0.001). Students who were not student leaders had a PTSD diagnosis more often than those who were student leaders. The marital status of parents was observed to have an effect on PTSD (χ^2^ = 11.309, *P* = 0.004), and students with single parents were more likely to suffer from PTSD than students with remarried and married parents.

### 3.7. Factors associated with PTSD

To investigate the predictors of total PTSD, univariate logistic regression analyses were first performed by entering each demographic variable individually into the regression model (Step 1). The results showed that approximately 33.2% of the sample (*n* = 2,068) met the diagnostic criteria for PTSD; seven items were significantly associated with PTSD among students, including education, age, grade, student leaders, living area, remarried, married, and education status of parents (*P* < 0.05). Middle school students were more vulnerable to suffering from PTSD than primary school students (OR = 1.497, 95%CI: 4.363–6.422). Adolescents were more likely to suffer from PTSD with age (OR = 1.143, 95%CI: 1.081–1.209). Compared to lower grades, adolescents with a traumatic history were more likely to develop PTSD at higher grades (OR = 1.102, 95% CI: 1.051–1.156). Student leaders tend to be less likely to have symptoms of PTSD than the general student (OR = 0.752, 95% CI: 0.631–0.896). Students living in urban areas were less likely to develop PTSD than those living in rural areas (OR = 0.742, 95% CI: 0.599–0.918). Students with remarried (OR = 0.493, 95%CI: 0.263–0.921) or married parents (OR = 0.407, 95% CI: 0.221–0.750) were less susceptible to PTSD than those with single parents. Participants with parents with a master's degree or above were found to have a lower risk of PTSD than participants with parents with less than a high school education (OR = 0.500, 95% CI: 0.273–0.913). There was no significant difference in cities of different socioeconomic levels, sex, and parental annual income (*P* > 0.05) (Table 7).

**Table 7:** Logistic regression analysis of the factors affecting total PTSD among children or adolescents (*N* = 2,068).

	**Univariable**			**Multivariable**		
	**Logistic regression**			**Logistic regression**		
**Variables**	**OR**	**95%CI**	***P***	**OR**	**95%CI**	***P***
**City**						
Small cities	1.000 (ref)					
Middle cities	0.895	0.722,1.109	0.311			
Big cities	0.96	0.779,1.184	0.705			
**Education**						
Elementary school student	1.000 (ref)			1.000 (ref)		
Middle school student	1.497	1.256, 1.784	0.000	1.290	1.016,1.637	0.037
**Age (years)**	1.143	1.081, 1.209	0.000	1.072	0.992,1.158	0.078
**Sex**						
Boy	1.000 (ref)					
Girl	1.001	0.840, 1.192	0.994			
**Grade**	1.102	1.051, 1.156	0.000			
**Living with their parents**						
No	1.000 (ref)					
Yes	0.769	0.581, 1.017	0.065			
**Student leaders**						
No	1.000 (ref)			1.000 (ref)		
Yes	0.752	0.631, 0.896	0.001	0.738	0.618,0.881	0.001
**Marital status of parents**						
Single	1.000 (ref)			1.000 (ref)		
Remarried	0.493	0.263, 0.921	0.027	0.474	0.252,0.893	0.021
Married	0.407	0.221, 0.750	0.004	0.420	0.227,0.778	0.006
**Living area**						
Rural	1.000 (ref)			1.000 (ref)		
Urban	0.742	0.599, 0.918	0.006	1.031	0.827,0.662	0.091
**Education status of parents**						
Less than high school	1.000 (ref)					
Vocational school	0.899	0.730,1.107	0.314			
College	0.932	0.717,1.212	0.600			
Master's degree or above	0.5	0.273,0.913	0.024			
**Annual** household **income ($)**	1	0.913, 1.094	0.992			

In multivariate regression model: Cox–Snell R^2^ = 0.024; Nagelkerke R^2^ = 0.032; Hosmer and Lemeshow test: chi-square = 19.177, P = 0.014.

Method = Backward Stepwise (Wald).

Grade and annual household income were not in the Equation.

All univariate variables that showed a relationship with the results were entered into the multivariate regression model (Step 2). Backward stepwise logistic regression was used to analyze the association between total PTSD and demographic variables. There were three variables associated with PTSD (*P* < 0.05), such as education, student leaders, and the marital status of parents. Compared with elementary school students, middle school students were more inclined to have PTSD (OR = 1.290, 95% CI: 1.016–1.637). Compared with the general students, student leaders were less inclined to have PTSD (OR = 0.738, 95% CI: 0.618–0.881). The parenting marital status had a significant effect on PTSD. Compared with the students with single parents, students with remarried or married parents were less inclined to have PTSD (the remarried: OR = 0.474, 95% CI: 0.252–0.893; the married: OR = 0.420, 95% CI: 0.227–0.778).

## 4. Discussion

### 4.1. Prevalence of traumatic events

The first aim of the study was to offer information on the prevalence of different types of traumatic events and the associated impact of traumatic events on children and adolescents. The results showed that nearly one-third of children or adolescents (34.3%) in Liaoning, China, had experienced at least one traumatic event, which is lower than 40.1% reported in the Chinese rural sample, 60.0% reported in the U.S. national sample, and 68.9% reported in the Mexican sample (4, 5, 8). The result was also lower than the Swedish study (63.0% of children and 89.5% of adolescents experienced a traumatic event) (3). The highest levels of traumatic events experienced by adolescents were found in the Palestinian (83.7%) and South African (99.7%) studies (6, 7). These differences in findings may be due in part to transcultural differences, natural conditions, and data collection.

The reported rates of witnessed trauma exceeded the rate of personally experienced trauma. Over 30 % of boys and girls had seen an industrial fire, explosion, or serious traffic accident (such as a car, airplane, or boat), which was the most traumatic experience witnessed by individuals in the sample. Approximately 20 % of students were exposed to the loss or sudden death of relatives, which was the most traumatic experience for the individuals in the sample, with more girls than boys experiencing such traumatic events.

The sudden death of a relative, industrial fire, explosion or serious traffic accident, and parental divorce were the three worst experiences reported by the participants. To our knowledge, 17.8% of the participants who were exposed to the worst traumatic event could cause a moderate and above burden. In addition, exposure to the most severe traumatic events was associated with impairment of somatic symptoms. Approximately 15.5–11.8% of the sample reported headache, dizziness, and rapid heartbeat, while diarrhea, tremors, and seizures were reported at lower rates. Over 30 % of the participants felt helpless, extremely fearful, and very tense or restless.

### 4.2. Prevalence of PTSD symptoms

The second aim of the study was to screen positive PTSD cases among children and adolescents who had experienced one or more traumatic events in their lifetimes. The findings revealed PTSD in 33.2% of children and adolescents exposed to traumatic events, which was 11.4% of the total sample. The prevalence of PTSD in our research was lower than participants who had experienced natural disasters. A meta-analysis study showed that 19.2, 30, 24.4, and 17.7% of children and adolescents suffered PTSD after earthquakes and floods over the first, second, third, and fourth 6-month periods (56). Exposure to natural disasters is associated with increased susceptibility to post-traumatic stress disorder in adolescents. This may be due to the fact that devastating natural disasters can have a devastating impact on individuals and communities, including those who are displaced, injured, or have lost loved ones or personal property (57, 58). In addition, several studies report a higher prevalence of PTSD (37.0–50.0%) in victims of interpersonal trauma (e.g., sexual violence and physical abuse) than in our study (30, 31). The excessive perception of life-threatening situations in children that result from interpersonal violence increases their risk of developing PTSD (48). Also, the prevalence of PTSD is even higher among children and adolescents who experienced war in Palestine (36%) and Syria (53%) (33, 59). This was probably because, children and adolescents may be exposed to challenges created by direct violence and non-violent impact due to war, such as seeing civilians killed, injured, or terrorized; being abducted, arrested, imprisoned; witnessing fierce conflicts by armed forces, artillery fire; and being forced to flee from their homes and lose their families. The wide range of results could be attributed to the type and degree of trauma exposure and the devastation of the event.

In a general community sample, our study reported a higher prevalence of PTSD (11.4%) than South African samples (4%) (32), US samples (4.7–8%) (48, 60), Malaysian samples (9.5%) and Indian samples (6.4%) (61). Many factors may contribute to cross-national differences in PTSD prevalence. One possibility to note is the psychiatric assessment instruments used. Participants in South Africa were evaluated and diagnosed according to DSM-IV criteria. A modified version of the Composite International Diagnostic Interview (CIDI) was administered by trained lay interviewers to US adolescents to assess their PTSD prevalence. The Harvard Trauma Scale was used to assess Malaysian and Indian students' PTSD prevalence. The second possibility is that there is increased awareness of PTSD among the general public and mental health services. Many students with PTSD may be afraid to recall or report traumatic events for fear of being stigmatized, and thus, the prevalence of PTSD is more likely to decrease (62). Also, differences in methodological approaches may account for this variability, including sample size, the use of different diagnostic criteria for PTSD, and the timing of the assessment after people have been exposed to a traumatic event (63).

### 4.3. Factors of PTSD

The study showed that students in middle school had more PTSD than in elementary school. Consistent with our research, studies of children following the earthquake found that older children were more susceptible to traumatic events than younger ones (64). A similar result was also found in tornado events (65). One explanation is that junior high school students were adolescents, a time when physical and emotional changes could lead to mental and physical instability, which increased the propensity for PTSD (6). Moreover, adolescence was considered to be a period of development associated with a high risk of exposure to negative or traumatic events and vulnerability (66). Furthermore, they had to deal with more stressful (such as learning pressure) and traumatic life events (67). Most traumatized students in China were reluctant to seek professional mental health services. When they are exposed to traumatic events, they may be prone to direct their pain, sadness, and other emotions internally, which may lead to the undesirable consequence of increasing the tendency to develop PTSD (68). It is therefore necessary for the government to train psychological counselors in schools and communities to assist traumatized children and adolescents.

The study demonstrated that student leaders were found to be an important protective factor for participants' PTSD. The reason for this may be that the success of student leaders in academic and interpersonal communication contributes to positive self-perceptions and emotional experiences at school and home. As excellent student representatives, most of them have a high level of psychological *suzhi*, which can effectively help students cope with stressful events and promote Mental Health (11, 69). The concept of psychological *suzhi* is a basic, stable, and internal psychological quality formed under the influence of congenital conditions, environment, and upbringing. Psychological *suzhi* is an endogenous factor affecting mental health. It is negatively correlated with depression and positively correlated with life satisfaction, subjective well-being, and positive mood (69, 70). In addition, student leaders with a good sense of confidence and security predict good peer relationships, which can mitigate and prevent the effects of stressful events (71–73). Therefore, school leaders and parents need to elevate adolescents' psychological *suzhi* in various ways.

Another factor that can affect the level of PTSD is the parents' marital status. In line with previous studies, students with a history of trauma whose parents remain married were less likely to develop PTSD than those whose parents are single. It can be speculated that greater marital quality may be under lower parenting stress due to more household income and greater social support (74). Parents who suffer from high levels of financial stress are unable to provide coping strategies and appropriate resources for the prevention of PTSD in children and adolescents who have experienced trauma (75). As a result, single-parent families lack the time and patience to nurture their children to satisfy their basic needs (76–78). Hence, a parent raising a child alone can worsen the effects of a traumatic event on the child in different ways. Confirming findings were also found in many studies that low socioeconomic status was more likely to develop PTSD (79, 80). Consequently, particularly counseling and professional support should be given to those students whose parents are single.

### 4.4. Strengths and limitations of the study

The results from the study had several limitations. First, the study relied on self-reported questionnaires, which may exist in social desirability bias and recall bias. Older students may be reluctant to disclose traumatic events that may raise sensitive issues, such as sexual abuse. Younger students may not be able to identify all traumatic experiences, such as being assaulted by a family member, especially those such as being physically attacked. Hence, face-to-face interviews and clinical assessments should be used in the future to more accurately diagnose children and adolescents with PTSD. Moreover, parental ratings may provide important information about children's functioning when young people are unreliable reporters of their behavior. Research has shown that children's PTSD is best assessed by utilizing information from multiple sources (81). Therefore, we call for more research to investigate the parents and teachers. Second, the survey used a cross-sectional design among children and adolescents in northern China. Thus, generalizations must be drawn with caution to other countries and other parts of China. Third, data were collected through the ETI-CA questionnaire and PTSD was assessed based on DSM-IV criteria. The epidemiology of children and adolescents with PTSD based on DSM-V criteria will need further investigation. In addition, the effects of traumatic event exposure may change over time due to the timing of the study endpoints, which may affect the generalizability of the findings.

Concerning the strengths, the study provides valuable evidence by using a large sample of children from families of different socioeconomic levels. Moreover, to thoroughly understand the research question, the measures of PTSD were widely applied and validated instruments. The parents may generally not be aware of all traumatic experiences, but they may also not be willing to disclose some events, particularly those such as child abuse, any mention of which may raise sensitive issues. This would result in selective underreporting of important events that could distort the results. However, both interpersonal and non-interpersonal events were reported with similar discrepancies between the samples.

## 5. Conclusion

Our study suggested that government, schools, teachers, experts, and parents should pay more attention to trauma exposure and post-traumatic stress disorder among a community sample of children and adolescents in China and provide emotional and psychological support. It is important to design appropriate emotional and psychological support into the intervention process to prevent trauma exposure and PTSD.

## Data availability statement

The datasets presented in this study can be found in online repositories. The names of the repository/repositories and accession number(s) can be found in the article/supplementary material.

## Ethics statement

Written informed consent was obtained from the minor(s)' legal guardian/next of kin for the publication of any potentially identifiable images or data included in this article.

## Author contributions

TY and LZ conceived and designed the research. TY, XianL, and LZ wrote the manuscript and analyzed the data. TY, XianL, HL, LZ, GX, J-lL, L-lG, LY, CW, DZ, YL, LS, XiaoL, and YH revised the manuscript. All authors reviewed the manuscript. All authors contributed to the article and approved the submitted version.

## References

[1] Association AP. Diagnostic and Statistical Manual of Mental Disorders. 4th ed. Washington, DC: American Psychiatric Publishing (1994).

[2] Association AP. Diagnostic and Statistical Manual of Mental Disorders, 5th Edition (DSM-5). Arlingon, VA: American Psychiatric Publishing (2013).

[3] Gustafsson PE Nilsson D Svedin CG. Polytraumatization and psychological symptoms in children and adolescents. Eur Child Adolesc Psychiatry. (2009) 18:274–83. 10.1007/s00787-008-0728-2 19156354

[4] Vacek S Whisman MA. Traumatic events and adolescent psychopathology in a United States national probability sample. Psychol Trauma. (2021) 13:277–83. 10.1037/tra0000961 32915042

[5] Orozco R Borges G Benjet C Medina-Mora ME López-Carrillo L. Traumatic life events and posttraumatic stress disorder among Mexican adolescents: results from a survey. Salud Publica Mex. (2008) 50 Suppl 1:S29–37. 10.1590/S0036-36342008000700006 18373005

[6] El-Khodary B Samara M Askew C. Traumatic events and PTSD among palestinian children and adolescents: the effect of demographic and socioeconomic factors. Front Psychiatry. (2020) 11:4. 10.3389/fpsyt.2020.00004 32296346 PMC7137754

[7] Closson K Dietrich JJ Nkala B Musuku A Cui Z Chia J. Prevalence, type, and correlates of trauma exposure among adolescent men and women in Soweto, South Africa: implications for HIV prevention. BMC Public Health. (2016) 16:1191. 10.1186/s12889-016-3832-0 27884181 PMC5123224

[8] Liang Y Zhou Y Liu Z. Traumatic experiences and posttraumatic stress disorder among Chinese rural-to-urban migrant children. J Affect Disord. (2019) 257:123–9. 10.1016/j.jad.2019.07.024 31301612

[9] Ponnamperuma T Sumathipala A Siribaddana S. Posttraumatic stress and co-occurrence of mental health problems in Sri Lankan adolescents. Asian J Psychiatr. (2020) 54:102444. 10.1016/j.ajp.2020.102444 33271723

[10] Vibhakar V Allen LR Gee B Meiser-Stedman R A. systematic review and meta-analysis on the prevalence of depression in children and adolescents after exposure to trauma. J Affect Disord. (2019) 255:77–89. 10.1016/j.jad.2019.05.005 31203106

[11] Heim C Nemeroff CB. The impact of early adverse experiences on brain systems involved in the pathophysiology of anxiety and affective disorders. Biol Psychiatry. (1999) 46:1509–22. 10.1016/S0006-3223(99)00224-3 10599479

[12] Malhi GS Das P Outhred T Irwin L Gessler D Bwabi Z. The effects of childhood trauma on adolescent hippocampal subfields. Aust N Z J Psychiatry. (2019) 53:447–57. 10.1177/0004867418824021 30712362

[13] Thomason ME Marusak HA Tocco MA Vila AM McGarragle O Rosenberg DR. Altered amygdala connectivity in urban youth exposed to trauma. Soc Cogn Affect Neurosci. (2015) 10:1460–8. 10.1093/scan/nsv030 25836993 PMC4631140

[14] Borges G Benjet C Orozco R Medina-Mora ME Mendez E Molnar BE. Traumatic life-events and suicidality among Mexican adolescents as they grow up: A longitudinal community survey. J Psychiatr Res. (2021) 142:171–8. 10.1016/j.jpsychires.2021.08.001 34359012

[15] Bynion T-M Cloutier R Blumenthal H Mischel ER Rojas SM Leen-Feldner EW. Violent interpersonal trauma predicts aggressive thoughts and behaviors towards self and others: findings from the National Comorbidity Survey-Adolescent Supplement. Soc Psychiatry Psychiatr Epidemiol. (2018) 53:1361–70. 10.1007/s00127-018-1607-x 30255381

[16] Shin SH Bouchard LM Montemayor B. An exploration of practitioners' perceptions and beliefs about trauma-informed youth drug prevention programs: a qualitative study. Prev Sci. (2022) 23:636–47. 10.1007/s11121-021-01300-0 34714501

[17] Ford JD Elhai JD Connor DF Frueh BC. Poly-victimization and risk of posttraumatic, depressive, and substance use disorders and involvement in delinquency in a national sample of adolescents. J Adolesc Health. (2010) 46:545–52. 10.1016/j.jadohealth.2009.11.212 20472211

[18] Battle DE. Diagnostic and statistical manual of mental disorders (DSM). CoDAS. (2013) 25:191–2. 10.1590/s2317-17822013000200017 24413388

[19] Lasiuk GC HK PTSD. Historical development of the concept erspectives in psychiatric care posttraumatic stress disorder part I: historical development of the concept. Perspect Psychiatr Care. (2006) 42:8. 10.1111/j.1744-6163.2006.00045.x 16480413

[20] Tang W Zhao J Lu Y Zha Y Liu H Sun Y. Suicidality, posttraumatic stress, and depressive reactions after earthquake and maltreatment: a cross-sectional survey of a random sample of 6132 chinese children and adolescents. J Affect Disord. (2018) 232:363–9. 10.1016/j.jad.2018.02.081 29510354

[21] Tang W Zhao J Lu Y Yan T Wang L Zhang J. Mental health problems among children and adolescents experiencing two major earthquakes in remote mountainous regions: a longitudinal study. Compr Psychiatry. (2017) 72:66–73. 10.1016/j.comppsych.2016.09.004 27744270

[22] Taukeni S Chitiyo G Chitiyo M Asino I Shipena G. Post-traumatic stress disorder amongst children aged 8-18 affected by the 2011 northern-Namibia floods. Jamba. (2016) 8:169. 10.4102/jamba.v8i2.169 29955304 PMC6014025

[23] Dyb G Jensen TK Nygaard E. Children's and parents' posttraumatic stress reactions after the 2004 tsunami. Clin Child Psychol Psychiatry. (2011) 16:621–34. 10.1177/1359104510391048 21565871

[24] Price M Yuen EK Davidson TM Hubel G Ruggiero KJ. Access and completion of a web-based treatment in a population-based sample of tornado-affected adolescents. Psychol Serv. (2015) 12:283–90. 10.1037/ser0000017 25622071 PMC4515396

[25] Ma EY Li FW. Developmental trauma and its correlates: a study of Chinese children with repeated familial physical and sexual abuse in Hong Kong. J Trauma Stress. (2014) 27:454–60. 10.1002/jts.21944 25158638

[26] Lee HB Shin KM Chung YK Kim N Shin YJ Chung US. Validation of the child post-traumatic cognitions inventory in korean survivors of sexual violence. Child Adolesc Psychiatry Ment Health. (2018) 12:32. 10.1186/s13034-018-0235-2 29946353 PMC6006562

[27] Tierens M Bal S Crombez G Loeys T Antrop I Deboutte D. Differences in posttraumatic stress reactions between witnesses and direct victims of motor vehicle accidents. J Trauma Stress. (2012) 25:280–7. 10.1002/jts.21692 22685086

[28] Avanci JQ Serpeloni F de Oliveira TP de Assis SG. Posttraumatic stress disorder among adolescents in Brazil: a cross-sectional study. BMC Psychiatry. (2021) 21:75. 10.1186/s12888-021-03062-z 33546640 PMC7866458

[29] Jenness JL Jager-Hyman S Heleniak C Beck AT Sheridan MA McLaughlin KA. Catastrophizing, rumination, and reappraisal prospectively predict adolescent PTSD symptom onset following a terrorist attack. Depress Anxiety. (2016) 33:1039–47. 10.1002/da.22548 27557454 PMC5325818

[30] Horowitz K McKay M Marshall R. Community violence and urban families: experiences, effects, and directions for intervention. Am J Orthopsychiatry. (2005) 75:356–68. 10.1037/0002-9432.75.3.356 16060732

[31] Paolucci EO Genuis ML Violato C A. meta-analysis of the published research on the effects of child sexual abuse. J Psychol. (2001) 135:17–36. 10.1080/00223980109603677 11235837

[32] Calitz FJW De Jongh NJ Horn A Nel ML Joubert G. Children and adolescents treated for post-traumatic stress disorder at the Free State Psychiatric Complex. Sout Afr J Psychiatry. (2014) 20:a441. 10.4102/sajpsychiatry.v20i1.441

[33] Agbaria N Petzold S Deckert A Henschke N Veronese G Dambach P. Prevalence of post-traumatic stress disorder among Palestinian children and adolescents exposed to political violence: a systematic review and meta-analysis. PLoS ONE. (2021) 16:e0256426. 10.1371/journal.pone.0256426 34437595 PMC8389374

[34] United United Nations Department of Economic and Social Affairs, Population Division. World Population Prospects 2022: Summary of Results (2022). UN DESA/POP/2022 /TR/NO. 3.

[35] National Bureau of Statistics of the People's Republic of China (2021). Available online at: https://data.stats.gov.cn/easyquery.htm?cn=C01&zb=A0301&sj=2021

[36] Ge L Xing C Li W. Trauma experiences and post-traumatic stress disorder prevalence in rural adolescents. Stud Psychol Behav. (2019) 17:395–401.

[37] Choi KR SJ Briggs EC Munro-Kramer ML Graham-Bermann SA Lee RC Ford JD. The dissociative subtype of posttraumatic stress disorder (PTSD) among adolescents: co-occurring PTSD, depersonalization/derealization, and other dissociation symptoms. J Am Acad Child Adolesc Psychiatry. (2017) 56:11. 10.1016/j.jaac.2017.09.425 29173740 PMC5726572

[38] Lewis SJ Arseneault L Caspi A Fisher HL Matthews T Moffitt TE. The epidemiology of trauma and post-traumatic stress disorder in a representative cohort of young people in England and Wales. The Lancet Psychiatry. (2019) 6:247–56. 10.1016/S2215-0366(19)30031-8 30798897 PMC6384243

[39] Herringa RJ. Trauma, PTSD, and the developing brain. Curr Psychiatry Rep. (2017) 19:69. 10.1007/s11920-017-0825-3 28823091 PMC5604756

[40] Bayer A Scott KM Koenen KC Aguilar-Gaxiola S Alonso J Angermeyer MC. Associations between lifetime traumatic events and subsequent chronic physical conditions: a cross-national, cross-sectional study. PLoS ONE. (2013) 8:e0080573. 10.1371/journal.pone.0080573 24348911 PMC3864645

[41] Zainudin NFB Ashari ZBM. A meta-analysis: the effects of child sexual abuse towards children. Asian Soc Sci. (20180 14:69. 10.5539/ass.v14n11p69

[42] Trickey D Siddaway AP Meiser-Stedman R Serpell L Field AP A. meta-analysis of risk factors for post-traumatic stress disorder in children and adolescents. Clin Psychol Rev. (2012) 32:122–38. 10.1016/j.cpr.2011.12.001 22245560

[43] Salmon K Bryant RA. Posttraumatic stress disorder in children. The influence of developmental factors. Clin Psychol Rev. (2002) 22:163–88. 10.1016/S0272-7358(01)00086-1 11806018

[44] Adams RE Boscarino JA. Predictors of PTSD and delayed PTSD after disaster: the impact of exposure and psychosocial resources. J Nerv Ment Dis. (2006) 194:485–93. 10.1097/01.nmd.0000228503.95503.e9 16840844 PMC2712250

[45] Garza K Jovanovic T. Impact of gender on child and adolescent PTSD. Curr Psychiatry Rep. (2017) 19:87. 10.1007/s11920-017-0830-6 28965303

[46] Tolin DF Foa EB. Sex differences in trauma and posttraumatic stress disorder: a quantitative review of 25 years of research. Psychol Bull. (2006) 132:959–92. 10.1037/0033-2909.132.6.959 17073529

[47] Turner HA Finkelhor D Ormrod R. Family structure variations in patterns and predictors of child victimization. Am J Orthopsychiatry. (2007) 77:282–95. 10.1037/0002-9432.77.2.282 17535126

[48] McLaughlin KA Koenen KC Hill ED Petukhova M Sampson NA Zaslavsky AM. Trauma exposure and posttraumatic stress disorder in a national sample of adolescents. J Am Acad Child Adolesc Psychiatry. (2013) 52:815–30.e14. 10.1016/j.jaac.2013.05.011 23880492 PMC3724231

[49] Yu H Nie C Zhou Y Wang X Wang H Shi X. Epidemiological characteristics and risk factors of posttraumatic stress disorder in chinese children after exposure to an injury. Disaster Med Public Health Prep. (2020) 14:486–93. 10.1017/dmp.2019.93 31610821

[50] Danielson CK Cohen JR Adams ZW Youngstrom EA Soltis K Amstadter AB. Clinical decision-making following disasters: efficient identification of PTSD risk in adolescents. J Abnorm Child Psychol. (2017) 45:117–29. 10.1007/s10802-016-0159-3 27103002 PMC5075270

[51] Milan S Zona K Acker J Turcios-Cotto V. Prospective risk factors for adolescent PTSD: sources of differential exposure and differential vulnerability. J Abnorm Child Psychol. (2013) 41:339–53. 10.1007/s10802-012-9677-9 22956298

[52] Memarzia J Walker J Meiser-Stedman R. Psychological peritraumatic risk factors for post-traumatic stress disorder in children and adolescents: a meta-analytic review. J Affect Disord. (2021) 282:1036–47. 10.1016/j.jad.2021.01.016 33601676

[53] Sadeghi K Poorolajal J Doosti-Irani A. Prevalence of modifiable risk factors of tuberculosis and their population attributable fraction in Iran: a cross-sectional study. PLoS ONE. (2022) 17:e0271511. 10.1371/journal.pone.0271511 35926063 PMC9352083

[54] Tagay S Düllmann S Hermans E Repic N Hiller R Senf W. The Essen Trauma-Inventory for children and adolescents (ETI-CA). Z Kinder Jugendpsychiatr Psychother. (2011) 39:323–40. 10.1024/1422-4917/a000126 21882155

[55] Jiang RF. The reliability and validity of the chinese version of essen trauma inventory for children adolescents. Master thesis. Huazhong University of Science and Technology (2011). Available online at: https://kns.cnki.net/KCMS/detail/detail.aspx?dbname=CMFD2012&filename=1012014668.nh

[56] Rezayat AA Sahebdel S Jafari S Kabirian A Rahnejat AM Farahani RH. Evaluating the prevalence of PTSD among children and adolescents after earthquakes and floods: a systematic review and meta-analysis. Psychiatric Quarterly. (2020) 91:1265–90. 10.1007/s11126-020-09840-4 32901423

[57] Schalinski I Rzeszutek M Dragan M Lis-Turlejska M Schier K Holas P. Exposure to self-reported traumatic events and probable PTSD in a national sample of Poles: why does Poland's PTSD prevalence differ from other national estimates? PLoS One. (2023) 18:0287854. 10.1371/journal.pone.0287854 37428736 PMC10332613

[58] Kemp AH Zheng Y Fan F Liu X Mo L. Life events, coping, and posttraumatic stress symptoms among chinese adolescents exposed to 2008 Wenchuan Earthquake, China. PLoS ONE. (2012) 7:29404. 10.1371/journal.pone.0029404 22295059 PMC3266232

[59] Kakaje A Al Zohbi R Alyousbashi A Abdelwahed RNK Hosam Aldeen O Alhalabi MM. Post-traumatic stress disorder (PTSD), anger and mental health of school students in Syria after nine years of conflict: a large-scale school-based study. Psychol Med. (2020) 52:1923–33. 10.1017/S0033291720003761 33267935

[60] Cloitre M. Over 60% of US adolescents have experienced a potentially traumatic event, almost 8% of whom have associated PTSD. Evid Based Ment Health. (2014) 17:27. 10.1136/eb-2013-101538 24167221 PMC13404688

[61] Redican E Vang ML Shevlin M Ghazali S Elklit A. The co-occurrence of potentially traumatic events (PTEs) and their associations with posttraumatic stress disorder (PTSD) in Indian and Malaysian adolescents. Acta Psychologica. (2023) 235:103896. 10.1016/j.actpsy.2023.103896 36990035

[62] Griffiths KM Christensen H. Internet-based mental health programs: a powerful tool in the rural medical kit. Aust J Rural Health. (2007) 15:81–7. 10.1111/j.1440-1584.2007.00859.x 17441815

[63] Bryant RA Nickerson A Morina N Liddell B. Posttraumatic stress disorder in refugees. Annu Rev Clin Psychol. (2023) 19:413–36. 10.1146/annurev-clinpsy-080921-080359 36854285

[64] Liu M Wang L Shi Z Zhang Z Zhang K Shen J. Mental health problems among children one-year after Sichuan earthquake in China: a follow-up study. PLoS One. (2011) 6:e14706. 10.1371/journal.pone.0014706 21373188 PMC3044135

[65] Xu W Yuan G Liu Z Zhou Y An Y. Prevalence and predictors of PTSD and depression among adolescent victims of the Summer 2016 tornado in Yancheng City. Arch Psychiatr Nurs. (2018) 32:777–81. 10.1016/j.apnu.2018.04.010 30201208

[66] Ogle CM Rubin DC Siegler IC. The impact of the developmental timing of trauma exposure on PTSD symptoms and psychosocial functioning among older adults. Dev Psychol. (2013) 49:2191–200. 10.1037/a0031985 23458662 PMC3806884

[67] Oransky M Hahn H Stover CS. Caregiver and youth agreement regarding youths' trauma histories: implications for youths' functioning after exposure to trauma. J Youth Adolesc. (2013) 42:1528–42. 10.1007/s10964-013-9947-z 23580028

[68] Chen SX Mak WW. Seeking professional help: etiology beliefs about mental illness across cultures. J Couns Psychol. (2008) 55:442–50. 10.1037/a0012898 22017551

[69] Pan Z Zhang D Hu T Pan Y. The relationship between psychological Suzhi and social anxiety among Chinese adolescents: the mediating role of self-esteem and sense of security. Child Adolesc Psychiatry Ment Health. (2018) 12:50. 10.1186/s13034-018-0255-y 30559836 PMC6292172

[70] Furlong MJ Gilman R Huebner ES. Handbook of Positive Psychology in Schools. Alexander PA, editor. New York, NY: Routledge (2014).

[71] Nyarko F Peltonen K Kangaslampi S Punamaki-Gitai RL. How stressful life events and violence are related to mental health: the protective role of social relations in African context. Heliyon. (2020) 6:e04629. 10.1016/j.heliyon.2020.e04629 32802978 PMC7419586

[72] Brown BB Lohr MJ. Peer-group affiliation and adolescent self-esteem: an integration of ego-identity and symbolic-interaction theories. J Pers Soc Psychol. (1987) 52:47–55. 10.1037/0022-3514.52.1.47 3820077

[73] Jiao C Wang T Liu J Wu H Cui F Peng X. Using exponential random graph models to analyze the character of peer relationship networks and their effects on the subjective well-being of adolescents. Front Psychol. (2017) 8:583. 10.3389/fpsyg.2017.00583 28450845 PMC5389982

[74] Kersh J Hedvat TT Hauser-Cram P Warfield ME. The contribution of marital quality to the well-being of parents of children with developmental disabilities. J Intellect Dis Res JIDR. (2006) 50:883–93. 10.1111/j.1365-2788.2006.00906.x 17100949

[75] Cross D Vance LA Kim YJ Ruchard AL Fox N Jovanovic T. Trauma exposure, PTSD, and parenting in a community sample of low-income, predominantly African American mothers and children. Psychol Trauma. (2018) 10:327–35. 10.1037/tra0000264 28481561 PMC5677577

[76] Parke RD Coltrane S Duffy S Buriel R Dennis J Powers J. Economic stress, parenting, and child adjustment in Mexican American and European American families. Child Dev. (2004) 75:1632–56. 10.1111/j.1467-8624.2004.00807.x 15566370

[77] Conger RD Wallace LE Sun Y Simons RL McLoyd VC Brody GH. Economic pressure in African American families: a replication and extension of the family stress model. Dev Psychol. (2002) 38:179–93. 10.1037/0012-1649.38.2.179 11881755

[78] Taylor ZE Larsen-Rife D Conger RD Widaman KF Cutrona CE. Life stress, maternal optimism, and adolescent competence in single mother, African American families. J Fam Psychol. (2010) 24:468–77. 10.1037/a0019870 20731493 PMC2928576

[79] Khamis V. Impact of war, religiosity and ideology on PTSD and psychiatric disorders in adolescents from Gaza Strip and South Lebanon. Soc Sci Med. (2012) 74:2005–11. 10.1016/j.socscimed.2012.02.025 22483708

[80] Kolltveit S Lange N II Thabet AA Dyregrov A Pallesen S Johnsen TB. Risk factors for PTSD, anxiety, and depression among adolescents in Gaza. J Traum Stress. (2012) 25:164–70. 10.1002/jts.21680 22522730

[81] Grant BR O'Loughlin K Holbrook HM Althoff RR Kearney C Perepletchikova F. A multi-method and multi-informant approach to assessing post-traumatic stress disorder (PTSD) in children. Int Rev Psychiatry. (2020) 32:212–20. 10.1080/09540261.2019.1697212 31880487 PMC7190440

